# Supplementary material for: Content-rich biological network constructed by mining PubMed abstracts
Source: BMC Bioinformatics. 2004 Oct 8;5:147. doi: 10.1186/1471-2105-5-147 (PMC528731; doi:10.1186/1471-2105-5-147)
Supplement: Additional File 2 — The original results of the above study (non-essential files are deleted to keep the file size under the limit set by BMC bioinformatics). [file 1471-2105-5-147-S2.bz2 › chilibotAdditionalFile2/dip10/145ID8955331E515/html/RSBV_RSBW.html]

 


 **RSBV** and **RSBW** 
  
Found 25 abstracts in PubMed, retrieved 10.  
 

 What does Google say? 
 PDF only 
| .edu only 

---

**Interactive relationship** (e.g. stimulation, inhibition, etc)

**Neutral relationship**- **RsbW**  is also a protein kinase which can phosphorylate  **RsbV** .  Ref: 12270815 J Bacteriol, 2002
- When cells are stressed,  **RsbW**  binds to unphosphorylated  **RsbV** , produced from the phosphorylated form of  **RsbV**  by two phosphatases RsbU and RsbP which are activated by stress.  Ref: 12270815 J Bacteriol, 2002
- According to the current understanding of sigmaB regulation, in unstressed cells the phosphorylated  **RsbV**  anti anti sigma is unable to complex the  **RsbW**  anti sigma, which is then free to bind and inactivate sigmaB.  Ref: 10632888 Mol Microbiol, 2000
- **RsbW** , purified as a dimer, forms complexes with  **RsbV**  and sigma B with different stoichiometries, i.e.,  **RsbW**  2  **RsbV**  2 and  **RsbW**  2 sigma B 1.  Ref: 12270815 J Bacteriol, 2002
- We conclude that the higher affinity of  **RsbW**  for  **RsbV**  than for sigma B, rather than a difference in the concentrations of  **RsbV**  and sigma B, is the driving force that is responsible for the switch of  **RsbW**  to unphosphorylated  **RsbV** .  Ref: 12270815 J Bacteriol, 2002

**Non-interactive relationship** (e.g. studied together, co-existance, homology, etc.)

- As determined by surface plasmon resonance, the dissociation constants of the  **RsbW**   **RsbV**  and  **RsbW**  sigma B interactions were found to be similar 63 and 92 nM, respectively.  Ref: 12270815 J Bacteriol, 2002
- The intracellular concentrations of  **RsbV** ,  **RsbW**  as a monomer, and sigma B measured before stress were similar 1..6, and 0.9 micro M, respectively.  Ref: 12270815 J Bacteriol, 2002
- The operon encoding the general stress transcription factor sigma B and two proteins of its regulatory network,  **RsbV**  and  **RsbW** , was cloned from the gram positive bacterium Bacillus anthracis by PCR amplification of chromosomal DNA with degenerate primers, by inverse PCR, and by direct cloning.  Ref: 10960085 J Bacteriol, 2000
- These two classes of stress are transmitted by separate signaling pathways which converge on the direct regulators of sigma B, the  **RsbV**  anti anti sigma factor and the  **RsbW**  anti sigma factor.  Ref: 11591687 J Bacteriol, 2001
- Evidence for gene identification includes the following i phylogenetic analyses of reported amino acid sequences for sigmaB and the closely related sigmaF proteins grouped L. monocytogenes sigmaB in the same cluster with the sigmaB proteins from Bacillus subtilis and Staphylococcus aureus, ii the gene order in the 68 bp portion of the L. monocytogenes sigB operon is rsbU  **rsbV**   **rsbW**  sigB rsbX and is therefore identical to the order of the last five genes of the B. subtilis sigB operon, and iii an L. monocytogenes sigmaB mutant had reduced resistance to acid stress in comparison with its isogenic parent strain.  Ref: 9658010 J Bacteriol, 1998
- The deduced amino acid sequences of orf2, orf3, orf4, and orf5 show 6 1, and 77% similarity to the B. subtilis proteins RsbU,  **RsbV** ,  **RsbW** , and SigB, respectively, with SigB representing the sigma factor and the Rsb proteins representing regulators of sigma B.  Ref: 9042755 Arch Microbiol< MedlineTA>Arch Microbiol, 1998
- The increase was greatest for  **RsbV** , whose concentration reached 13 micro M, while those of  **RsbW**  as a monomer and sigma B reached 11.8 and 4.9 micro M, respectively.  Ref: 12270815 J Bacteriol, 2002
- Nonetheless, an analysis of the complexes by nondenaturing polyacrylamide gel electrophoresis in competition assays suggested that the affinity of  **RsbW**  2 for  **RsbV**  is much higher than that for sigma B.  Ref: 12270815 J Bacteriol, 2002
- Bracketing slr1860 icfG are i ORF slr1861, whose predicted product resembles the SpoIIAB, RsbT, and  **RsbW**  protein kinases from B. subtilis, and ii ORFs slr1856 and slr1859, whose predicted products resemble the respective phosphoprotein substrates for the B. subtilis protein kinases SpoIIAA, RsbS, and  **RsbV** .  Ref: 10438742 J Bacteriol, 1999
- sigma B activity is regulated post translationally by a multi component network composed of two coupled partner switching modules, RsbX RsbS RsbT and RsbU  **RsbV**   **RsbW** , each containing a serine phosphatase X or U, an antagonist protein S or V, and a switch protein serine kinase T or W.  Ref: 9179850 Mol Microbiol, 1997
